# Desiderosmia-Associated Iron Deficiency: A Case Report and Review of the Literature

**DOI:** 10.7759/cureus.106380

**Published:** 2026-04-03

**Authors:** Aaron Lee, Shantel Morales, Elisa Quiroz

**Affiliations:** 1 Internal Medicine, San Ysidro Health, San Ysidro, USA; 2 Hematology, San Ysidro Health, San Ysidro, USA

**Keywords:** clinical manifestation, desiderosmia, iron-deficiency, iron deficiency anemia (ida), smell craving

## Abstract

Iron deficiency is the most common cause of anemia worldwide, yet desiderosmia remains rarely described as one of its manifestations. The term "desiderosmia" was first proposed in a case series by Hansen et al. to describe compulsive olfactory cravings for specific scents observed in three symptomatic patients with iron deficiency, derived from the Latin word “desiderare” for desire and the Greek word “osme” for smell. Desiderosmia appears to be closely related to pica, a common manifestation of iron deficiency, though the key difference is that patients report olfactory cravings without the desire to consume the scent-producing substance. As far as the authors of this report are aware, the number of published cases of desiderosmia is extremely scarce, and the term itself has yet to become recognized in medical literature, owing to its novelty. We report the case of a 32-year-old woman with menorrhagia and iron deficiency anemia (IDA) refractory to oral ferrous sulfate despite adherence. Despite her hemoglobin improving from 9.1 to 9.9 g/dL (reference range: 12-15.5 g/dL), her ferritin remained critically low at 1 ng/mL (reference range: 30-400 ng/mL). At that time, she developed an intense urge to smell pine-scented cleaning products, alongside migraines and restless leg syndrome, hypothesized to be neuropsychological symptoms linked to altered CNS iron homeostasis. We speculate that desiderosmia may share a similar neurological mechanism, correlating more closely with the severity of iron store depletion rather than with anemia severity. Recognition of this symptom prompted identification of IDA not improving with oral iron and escalation of therapy to IV ferrous carboxymaltose. Following treatment with intravenous iron repletion, her ferritin increased to 52 ng/mL, and she no longer experienced desiderosmia or any other iron deficiency symptoms. This case highlights the need for validating studies of desiderosmia's potential as a diagnostic marker in cases of severe iron deficiency and the symptom's pathophysiology.

## Introduction

Iron deficiency anemia (IDA)

IDA is characterized by reduced hemoglobin due to insufficient iron stores. Anemia affects nearly 30% of the global population, with iron deficiency accounting for roughly half of cases, approximately 1.2 billion people worldwide [1,2]. In the United States, iron deficiency occurs in 9%-12% of women and up to 20% of those of reproductive age [3]. Higher incidence has also been reported in Hispanic women [2]. Notably, iron deficiency without anemia is at least twice as prevalent as IDA, underscoring the substantial subclinical burden [4]. The widespread impact of iron deficiency and IDA is clinically significant, as many patients can develop debilitating fatigue, impaired cognition, and risks to neonatal development during pregnancy. 

Etiologies include blood loss, dietary insufficiency, malabsorption, chronic inflammation, and infection [4]. At-risk populations for iron deficiency include pregnant or menstruating individuals, young children, and those with inflammatory bowel disease or chronic kidney disease [2]. For these higher-risk patients, maintaining a high index of suspicion for iron deficiency is crucial. Recognizing iron deficiency is important not only for treating symptomatic anemia, if also present, but also to prompt further investigation of serious underlying causes such as gastrointestinal blood loss or colorectal cancer. 

Diagnosis requires recognizing clinical manifestations and ordering targeted laboratory testing. Common symptoms include fatigue, restless leg syndrome, hair loss, headache, and pica [4]. When suspicion is sufficient, initial workup screens for anemia, defined by the World Health Organization as hemoglobin <12.0 g/dL in non-pregnant adult women [1]. Iron deficiency is further characterized by low serum iron (typically <60 µg/dL) and reduced ferritin, though the defining threshold varies by society: the American Gastroenterological Association recommends ≤45 ng/mL, while the American Society of Hematology defines deficiency as ≤30 ng/mL in the general population, with consideration of ≤50 ng/mL in high-risk individuals [5,6]. As anemia and iron deficiency worsen, patients report common manifestations as described above, but may also experience desiderosmia. 

Desiderosmia

Despite the high global prevalence of IDA, one of its most underrecognized manifestations is desiderosmia, an intense craving for specific odors of inedible substances such as rubber, menthol, gasoline, bleach, and cleaning products. Desiderosmia can be described as an olfactory counterpart to pica, but distinguishes itself as patients do not experience a desire to consume the scent-producing substance that is craved. First introduced by Hansen et al., the term combines the Latin desiderare (to desire) and the Greek osmē (smell) to characterize unusual scent cravings observed in iron-deficient patients [7]. At the present time, there is an absence of a formally recognized diagnostic label for this symptom in standard medical nomenclature. 

The existing literature consists largely of case reports, most documenting transient relief upon scent exposure and prompt resolution following iron repletion, particularly after intravenous supplementation. This consistent pattern implicates iron deficiency as the physiologic driver rather than a coincidental behavioral phenomenon. Hansen et al. hypothesized that desiderosmia is related to pica in that both arise in the setting of profound iron depletion [7]. Subsequent reports by Yanardag and Harris et al. corroborated this association, documenting odor cravings in patients with markedly low ferritin (<6 ng/mL) that resolved with iron repletion, further distinguishing the symptom from primary psychiatric or behavioral causes [8,9]. Collectively, these cases all describe symptom manifestation at a ferritin threshold below 10 ng/mL, suggesting desiderosmia reflects severe iron depletion. This deficiency may alter central neurotransmission or sensory processing, which would be consistent with the proposed pathophysiology of better-studied neurobehavioral manifestations of iron deficiency, such as pica and restless leg syndrome [3].

Pathophysiology and prevalence 

The pathophysiology of desiderosmia is not well understood, but it is plausible that the neurobiologic basis likely overlaps with that of pica, given the inherent sensory interplay between taste and olfaction [9,10]. Iron deficiency is thought to disrupt dopaminergic transmission within mesolimbic reward pathways, driving compulsive pica cravings such as pagophagia (ice) and geophagia (dirt) [3]. Beyond dopamine, iron is also essential for myelination, monoamine metabolism, and neuronal energy production; experimental models confirm that depleted CNS iron impairs neurotransmitter synthesis and alters sensory processing [10]. Its association with restless legs syndrome, migraines, and other sensorimotor disturbances further supports a neurologic basis for iron-related behavioral symptoms. Some authors propose that desiderosmia represents a variant within this broader neurobehavioral spectrum, potentially emerging once ferritin falls below a critical threshold [11]. However, the evidence base remains limited to isolated case reports and small series, constraining broader conclusions about prevalence and pathophysiology.

The apparent rarity of desiderosmia can be explained by the combination of a limited patient population reaching such profound ferritin depletion and underreporting driven by a lack of clinician inquiry during evaluation. Targeted questioning may therefore uncover a higher prevalence than currently recognized, positioning desiderosmia as a potential clinical marker of severe iron deficiency. This case report aims to highlight desiderosmia as an emerging manifestation of IDA encountered in the outpatient setting and to advocate for further research into its underlying mechanisms, diagnostic utility, and role in guiding clinical management.

## Case presentation

The patient was a 32-year-old woman with a history of menorrhagia and chronic migraine. She was first diagnosed with IDA during her first pregnancy and was subsequently started on daily oral ferrous sulfate. However, she self-discontinued iron supplementation postpartum. Unfortunately, the patient was then lost to follow-up with primary care. Two years later, she returned to her primary care physician with symptoms of fatigue, headaches, and brittle nails. She continued to have heavy menstrual periods at that time. Laboratory evaluation revealed hemoglobin of 9.1 g/dL (reference range: 12-15.5 g/dL), total iron of 13 mcg/dL (reference range: 50-170 mcg/dL), iron saturation of 3% (reference range: 20%-50%), and ferritin of 5 ng/mL (reference range: 30-400 ng/mL). Based on findings consistent with severe iron deficiency, the patient was restarted on oral ferrous sulfate 325 mg every other day and referred to obstetrics and gynecology for menorrhagia. 

**Figure 1:**
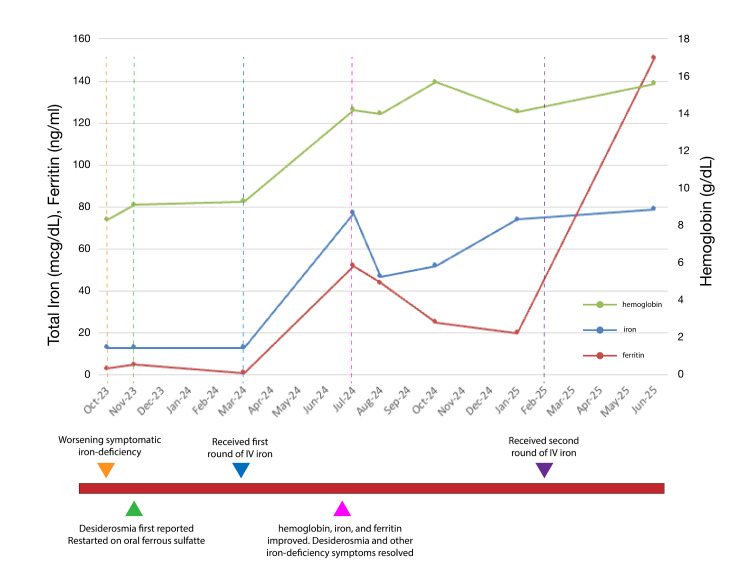
Timeline of tracked iron deficiency anemia biomarkers vs. symptom manifestation Serial hemoglobin (g/dL, right axis), serum iron (mcg/dL, left axis), and ferritin (ng/mL, left axis) are plotted from October 2023 through June 2025, with dashed vertical lines marking key clinical events. Desiderosmia was first reported in November 2023, with serum ferritin critically low at <1 ng/mL despite resumption of oral ferrous sulfate. Symptoms resolved and ferritin improved following the first intravenous (IV) iron course in March 2024. Notably, desiderosmia did not recur when ferritin was approximately 20 ng/mL in January 2025, suggesting a possible threshold below which the symptom emerges.

Despite three months of daily oral iron supplementation and resolution of heavy menstrual bleeding with placement of an intrauterine device, she reported worsening fatigue and dyspnea on exertion, so she was referred to hematology. Additional evaluation, including a colonoscopy, celiac testing, and vitamin deficiency screening, was negative. The only medications taken by the patient were prescribed ferrous sulfate and an over-the-counter vitamin B complex. Figure 1 describes the patient's developing iron-deficiency symptoms with her correlating relevant bloodwork. Her laboratory findings on initial hematology consultation revealed slightly improved hemoglobin of 9.9 g/dL, stable iron of 13 mcg/dL, and iron saturation of 3%, but worsening ferritin of 1 ng/mL. An alternate oral iron dosing regimen, to be taken every other day, was recommended to enhance absorption by avoiding hepcidin-mediated inhibition. After three months of strict adherence to this regimen, her ferritin level remained critically low at 1 ng/mL, and the patient began reporting hair loss, lightheadedness, and restless legs. Additionally, she endorsed a new intense craving to smell pine-scented cleaning fluid, fitting the description of desiderosmia, a novel neurobehavioral manifestation of iron deficiency, to compulsively seek out and smell specific scents. Her scent-seeking behavior occurred randomly, one to two times per day, several times a week, sometimes awakening her from sleep to actively search for pine-scented substances. The patient did not have any known psychiatric or behavioral disorders and did not report any prior environmental exposure triggers.

Given the failure of oral iron supplementation to increase ferritin levels and the emergence of additional IDA symptoms, she was transitioned to intravenous iron replacement. Upon receiving two doses of 750 mg intravenous ferric carboxymaltose spaced seven days apart, her iron improved to 77 mcg/dL, 27% iron saturation, and a ferritin of 52 ng/mL. Her anemia also resolved with her hemoglobin increasing to 13.2 g/dL. Four months after intravenous iron therapy, the patient reported complete resolution of her craving for pine scents and improvement in her other IDA symptoms. The patient maintained her ferritin levels and remained anemia-free with interval iron infusions. Interestingly, desiderosmia did not recur despite biochemical recurrence of iron deficiency (ferritin was 20 ng/mL, iron saturation 21%, and serum iron 74 mcg/dL), suggesting that desiderosmia may be more closely associated with severe iron deficiency.

## Discussion

Desiderosmia, a compulsive craving for specific odors, is an underrecognized sensory manifestation of iron deficiency, described far less frequently than pica or restless leg syndrome. Its pathophysiology remains poorly defined but is hypothesized to involve iron-dependent alterations in neural reward pathways, limbic system function, or olfactory bulb metabolism, all of which are sensitive to iron availability [12]. Iron is a critical cofactor for tyrosine hydroxylase and other enzymes involved in neurotransmitter synthesis; deficiency may therefore dysregulate reward processing and sensory perception, driving compulsive olfactory cravings [13]. Additionally, structural and functional CNS changes associated with iron deficiency may further heighten sensory-seeking behaviors [14]. However, it is important to note that while our patient did not have a known psychiatric or behavioral disorder, a formal evaluation was not completed, and so such a psychiatric contribution to her desiderosmia cannot be fully excluded.

Desiderosmia's close link to pica, both compulsive, non-nutritive cravings, suggests a shared neurobiologic pathophysiology. Prior studies of pica indicate that iron deficiency may be the cause of disruption to dopaminergic and serotonergic pathways, leading to abnormal craving to consume non-nutritive substances [15]. Given the intrinsic connection between the gustatory and olfactory systems, desiderosmia is hypothesized to reflect the same process, expressed through scent rather than taste. This was observed in our patient, who developed desiderosmia concurrently with worsening migraines and restless leg syndrome, possibly supporting iron's role as a depleted cofactor across multiple neurobehavioral symptoms, including this novel manifestation. In a large outpatient series, both pica and restless leg syndrome improved with iron repletion, reinforcing that such behaviors could represent physiologic responses to impaired CNS iron homeostasis [16]. As a proposed hypothesis of pathophysiology, it rests largely on observational associations and is limited by the lack of mechanistic testing. 

Desiderosmia manifestation at critically low ferritin levels and resolution once iron was adequately repleted was seen in both this case and several others (Table 1). The symptom appears to be closely tied to the severity of iron store depletion, which may suggest a neurologic threshold effect analogous to other iron-deficiency manifestations. Restless leg syndrome has been observed disproportionately at ferritin levels <50-75 ng/mL and pica typically below 10-15 ng/mL [17,18]. It has been theorized that symptoms emerge once iron stores fall below the threshold required to maintain normal neurologic function, as seen with iron-associated basal ganglia dysfunction in restless leg syndrome [19]. However, interpretation is limited by the fact that serum ferritin may not reliably reflect iron availability within the CNS, leaving a threshold undefined. While desiderosmia has been mentioned in limited case reports, no ferritin threshold for its occurrence has been established [7-9,12,20]. Published cases report ferritin levels ranging from 1-6 ng/mL (Table 1), and our patient did not develop a craving for pine-scented products until her ferritin reached 1 ng/mL, suggesting a possible threshold below that seen with pica. This was emphasized by the fact that our patient did not have a recurrence of desiderosmia despite her ferritin being at 20 ng/mL. It may also be possible that desiderosmia manifests as an iron depletion severity-dependent phenomenon rather than a fixed range. 

**Table 1:** Cases of desiderosmia odor cravings and described ferritin levels

Patient	Author	Year	Craving (Odor)	Ferritin level	Resolution after iron therapy (Y/N)
1	Current case	2026	Pine-scented cleaning products	1 ng/mL	Y
2	Hansen et al. [7]	2017	Gasoline fumes	Not reported	Y
3	Hansen et al. [7]	2017	Charcoal	Not reported	Y
4	Hansen et al. [7]	2017	Cleaning products (bleach/ammonia)	Not reported	Y
5	Yanardag et al. [8]	2019	Exhaust fumes, gasoline	2.5 ng/mL	Y
6	Yanardag et al. [8]	2019	Methanol	5 ng/mL	Y
7	Harris et al. [9]	2022	Lemon cleaning product	<6 ng/mL	Y
8	Harris et al. [9]	2022	Bodywash	<6 ng/mL	Y
9	Sharma et al. [20]	2008	Ammonia	Not reported	Not reported
10	Kettaneh et al. [12]]	2005	Gasoline/solvents	Not reported	Y
Note: ng/mL = nanogram/milliliter; Y = yes, N = no					

Several limitations complicate extrapolating a ferritin threshold for desiderosmia. Many reported cases lack documented ferritin levels, odor descriptions are subjective and culturally influenced, and reporting bias toward unusual cases may skew observed values. Ferritin monitoring was also inconsistent across reports, including this case. Further prospective studies, neurobiological investigation, and standardization of diagnostic criteria are needed to determine whether a reproducible threshold exists and whether desiderosmia could serve as a reliable clinical marker of iron deficiency severity.

## Conclusions

Recognition of desiderosmia may carry practical clinical implications but still requires validation. If olfactory cravings are found to be diminished with iron repletion, their resolution could serve as an early patient-reported marker of therapeutic efficacy. Particularly where laboratory follow-up is delayed or access is limited. Conversely, persistence or recurrence may signal ongoing blood loss, malabsorption, or non-adherence to treatment. Given that iron deficiency remains one of the most common nutritional deficiencies, even rare manifestations may affect a substantial number of individuals. Increased clinician awareness and targeted questioning about unusual sensory cravings, including smells, may help improve detection of severe or refractory cases. This case highlights a possible pathophysiologic mechanism by which iron deficiency disrupts neurologic pathways driving compulsive olfactory behavior and raises the possibility of a ferritin threshold-dependent manifestation that warrants further exploration in the context of diagnosis and management. Future prospective studies are needed to establish prevalence, define the neurobiologic mechanisms, identify ferritin thresholds associated with symptom onset, and evaluate desiderosmia's potential as a reliable clinical marker of iron deficiency severity.
